# The Gracilis Myocutaneous Free Flap: A Quantitative Analysis of the Fasciocutaneous Blood Supply and Implications for Autologous Breast Reconstruction

**DOI:** 10.1371/journal.pone.0036367

**Published:** 2012-05-09

**Authors:** Iain S. Whitaker, Maria Karavias, Ramin Shayan, Cara Michelle le Roux, Warren M. Rozen, Russell J. Corlett, G. Ian Taylor, Mark W. Ashton

**Affiliations:** 1 The Taylor Lab, Department of Anatomy and Neurosciences, University of Melbourne, Parkville, Victoria, Australia; 2 Swansea University College of Medicine, Swansea University, Swansea, Wales, United Kingdom; Harvard Medical School, United States of America

## Abstract

**Background:**

Mastectomies are one of the most common surgical procedures in women of the developed world. The gracilis myocutaneous flap is favoured by many reconstructive surgeons due to the donor site profile and speed of dissection. The distal component of the longitudinal skin paddle of the gracilis myocutaneous flap is unreliable. This study quantifies the fasciocutaneous vascular territories of the gracilis flap and offers the potential to reconstruct breasts of all sizes.

**Methods:**

Twenty-seven human cadaver dissections were performed and injected using lead oxide into the gracilis vascular pedicles, followed by radiographic studies to identify the muscular and fasciocutaneous perforator patterns. The vascular territories and choke zones were characterized quantitatively using the ‘Lymphatic Vessel Analysis Protocol’ (LVAP) plug-in for Image J® software.

**Results:**

We found a step-wise decrease in the average vessel density from the upper to middle and lower thirds of both the gracilis muscle and the overlying skin paddle with a significantly higher average vessel density in the skin compared to the muscle. The average vessel width was greater in the muscle. Distal to the main pedicle, there were either one (7/27 cases), two (14/27 cases) or three (6/27 cases) minor pedicles. The gracilis angiosome was T-shaped and the maximum cutaneous vascular territory for the main and first minor pedicle was 35×19 cm and 34×10 cm, respectively.

**Conclusion:**

Our findings support the concept that small volume breast reconstructions can be performed on suitable patients, based on septocutaneous perforators from the minor pedicle without the need to harvest any muscle, further reducing donor site morbidity. For large reconstructions, if a ‘T’ or tri-lobed flap with an extended vertical component is needed, it is important to establish if three territories are present. Flap reliability and size may be optimized following computed tomographic angiography and surgical delay.

## Introduction

Breast cancer is the leading cause of cancer death in females worldwide [1]–[3], accounting for 23% (1.38 million) of the total new cancer cases and 14% (458,400) of the total cancer deaths in 2008 [4]. Hundreds of thousands of women are diagnosed with breast cancer each year in the western world (83·2 new breast cancers per 100,000 population) and almost half of those treated have a mastectomy [5], [6]. Breast reconstructions are often necessary to replace the breast and improve quality of life. In 2010, 61·5 mastectomies were performed per 100,000 people in the developed world (*OECD*), making it one of the most common contemporary surgical procedures [2].

In 2009, over 86,000 breast reconstructions were performed in the United States alone, a statistic which is increasing [7]. A substantial proportion of these comprised autologous reconstructions, with autologous flaps felt to offer the most natural looking and feeling breast. Although the American Society of Plastic Surgeons (ASPS) procedural statistics in 2010 show that implant based breast reconstruction remains the most practiced form of breast reconstruction in the United States (77%), this statistic is felt to be changing [8], [9], particularly as the relative increase in cost is worthwhile when considering reported superior outcomes and patient satisfaction [9], [10]. Other influences on the changing landscape of breast reconstruction includes the increasing media attention and reports of implant complications, such as rupture, capsular contracture or anaplastic large cell lymphoma (ALCL) [11]–[14].

Bilateral breast reconstruction procedures currently constitute around 10% of breast cancer operations and are becoming increasingly frequent with the greater use of BRCA gene testing and breast magnetic resonance imaging (MRI) [15]–[17] which increases the need for improved reconstructive options.

There are many options for in flap selection for autologous breast reconstruction, a decision based on open discussion between surgeon and patient. Free autologous options are commonly used [18] and include abdominal based flaps such as deep inferior epigastric artery perforator (DIEP) [19], transverse rectus abdominis myocutaneous (TRAM) [20] and superficial inferior epigastric artery (SIEA) [21] flaps, gluteal based flaps such as superior gluteal artery perforator (SGAP) and inferior gluteal artery perforator (IGAP) flaps [22], flaps from the trunk such as latissiums dorsi (LD) [23] and thoracodorsal artery perforator (TAP/TDAP) flaps [24], [25] and flaps from the lower limb such as gracilis [26]–[30] and less commonly anterolateral thigh (ALT) flaps [31]. Advances in reconstructive surgery are based on the search for autologous tissue with minimal donor site morbidity. Sometimes this necessitates a reappraisal of flaps previously considered difficult or unreliable such as the longitudinal gracilis skin paddle.

The gracilis myocutaneous flap is favoured by several influential groups of reconstructive surgeons due to the donor site profile and speed of dissection and has traditionally been used to reconstruct small to medium sized breasts [26]. It is particularly attractive to patients who do not want abdominal, buttock or back scars. Donor site scars are well concealed and it gives the added benefit of a thigh lift. There are several key benefits of this flap over larger musculocutaneous flaps such as the latissimus dorsi flap – the donor site is potentially better, with no extra incisions on the trunk and no sacrifice of a major functional muscle and there is less muscle bulk which can influence the early aesthetic outcome and make late results difficult to interpret early. The work described in this paper, and that of others offers the exciting potential to reconstruct breasts of all sizes, and open up this reconstructive option and minimal donor site morbidity to a wider group of women worldwide.

### The Gracilis Myocutaneous Flap

The use of the gracilis muscle in reconstructive surgery was first described by Pickrell in 1952 for rectal sphincter reconstruction [32]. Use of the musculocutaneous variant was first reported by Orticochea [33] in 1972 as a cross-legged flap, before McCraw used a pedicled gracilis musculocutaneous flap for vaginal reconstruction in 1976 [34]. In the same year Harii et al. reported the first gracilis free flap for reconstruction of the head and neck and lower extremity [35]. Since the late seventies the gracilis myocutaneous flap has been used for soft tissue reconstruction of the breasts [27]–[30], [36], [37], head and neck [38]–[40], upper and lower extremity [41], [42] and the genitoperineal region [43], [44]. It has also been used as a free functioning muscle transfer for restoration of elbow flexion [45]–[47], dynamic analplasty [48], [49] and facial reanimation [50]–[52].

Several authors have described varying degrees of distal cutaneous necrosis when a longitudinal skin paddle is used [34], [53], and contemporary surgeons still regard the distal skin component of the longitudinal design as unreliable. There is a degree of contention in the literature regarding the most reliable flap design, dimensions and operative technique. In 1992, Yousif et al. made a major advance by performing anatomical injection studies that formed the basis for modifying the traditional longitudinally oriented gracilis musculocutaneous flap to a transverse design [54]. The transverse myocutaneous (or upper) gracilis (TMG or TUG) flap has since gained popularity, particularly in Europe, because of its advantages over the longitudinal design including the reliability of the proximal skin paddle and faster dissection times. The main criticism of the TMG/TUG flap is the lack of volume. Several authors have made attempts to modify the design to increase flap volume [26]–[28], [30], [55]–[57] **(** **Table 1** **)**. The heaviest flaps reported to date have been 576 g [57] and the largest skin paddles 30 cm×10 cm [30].

**Table 1:** Modifications of gracilis flap design in order to increase flap volume.

Year	Author	Technique
2004, 2008	Schoeller^26,28^	Harvesting the flap posteriorly
2009	Vega^54^	Inclusion of both vertical and transverse components (‘L’ shaped design)
2009	Peek^53^	Inclusion of the angiosomes of the major and minor pedicles (vertical extended gracilis perforator flap)
2010	Fattah^55^	Inclusion of the superficial thigh fat distal to the inferior border of the transverse skin paddle
2011, 2012	Saint Cyr^24,25^	Including an extended transverse and vertical component (‘Trilobed’ variant)

### Clarification of Vascular Nomenclature

**Figure 1:**
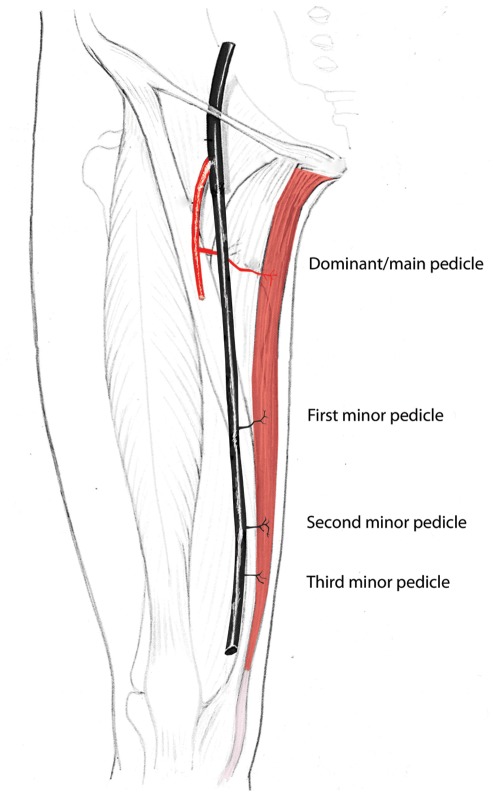
Blood supply to the gracilis flap clarifying the nomenclature to be used in this article, and showing the dominant and minor pedicles. (Adapted from Strauch B, Han-Liang Yu. (2006) *Atlas of Microvascular Surgery*, 2^nd^ Edition, Thieme).

The gracilis muscle is a type II muscle according to the scheme of Mathes and Nahai [58]. Usually two or three vascular pedicles, accompanied by paired venae comitantes, enter the muscle on its deep surface and nourish it **(** **Figure 1** **)**. Of these, the proximal pedicle is generally the dominant blood supply to the muscle, originating from the profunda femoris vessels and passing between the adductor longus and adductor brevis before reaching the gracilis muscle hilum. The distal vascular pedicles arise from the superficial femoral vessels. The *dominant* vascular supply to the gracilis is the proximal vascular pedicle [59] and supplies approximately 70 % of the bulk of the muscle [60]. Located between the adductor longus and adductor brevis muscles, it is accompanied by two venae comitantes of similar size [54], [60] and enters the gracilis 10±2 cm distal to its attachment at the pubic tubercle [53], [54]. Although reports [33], [35], [61] describe the origin of the dominant pedicle to be directly from the profunda femoris or medial circumflex femoral artery, findings from other studies present the adductor artery as an alternative origin of this pedicle [53], [62]–[64]. The dominant pedicle divides upon entering the gracilis muscle, passing proximally and distally parallel to the longitudinal muscle fibres [60], before anastomosing with branches of the second (minor) pedicle [60], [65], [66]. The dominant pedicle gives off perforating vessels to the skin overlying the proximal third of the gracilis muscle [54], [61], [67], [68]. The number of minor (distal) pedicles range from one to five [60]–[63], [65], [66], which distally supply 15% to 30% of the bulk of the muscle [60]. The first distal pedicle tends to enter the muscle 22 to 27 cm distal to the ischiopubic attachment [55]. Although reports allude to their superficial femoral artery origin, this varies in the most distal minor pedicle [61], [63], [65], [66]. A small minor pedicle that is proximal to the main pedicle has been reported [69], [70]. For the purposes of this study, it will be known as the proximal accessory pedicle. This is not always present and varies in its origin.

### Fasciocutaneous Supply

Over the past four decades, the accrued knowledge of the vascular anatomy of the gracilis muscle has emphasized its consistent and reliable neurovascular anatomy. The elucidation of the vascular territory of the overlying skin is not as extensive. The traditional skin paddle was longitudinal, until Yousif showed that the perforator vessels of the main pedicle had a marked tendency to travel in a transverse direction [71]. The discovery of the transverse tendency of these perforators led to the design of the transverse musculocutaneous (upper) gracilis flap (TMG/TUG) in which the skin island is centered over the main (dominant) pedicle and follows the direction of the cutaneous perforators. Kappler et al. further elucidated the number and localization of proximal cutaneous perforator vessels in a cadaveric study of the gracilis [67]. Their external diameter was between 0.5 and 1 mm and perforator diameter was inversely proportional to the number of perforators identified per pedicle. In the TUG flap, the perforators were localized within a 6×6 cm^2^ area from the entrance point of the proximal pedicle. They exited the gracilis opposite to the entry point of the main pedicle. The cutaneous distribution of the perforators of the main gracilis pedicle has been described following selective ink injections into the dominant pedicle [61], [63]. The dominant pedicle angiosome was oval shaped with skin paddle dimensions ranging from 11×8 cm to 16.5×11 cm with the largest and most numerous cutaneous perforators present in the proximal third. In previous studies, musculocutaneous perforators of the dominant pedicle dominated in number and diameter and were more consistent in their anatomy compared with septocutaneous perforators. Peek et al. found an average of five perforators per thigh in an anatomical study of 43 cadavers [55] and seventy-two percent of these were musculocutaneous. Yousif reported 60% of main pedicle perforators were musculocutaneous in 20 cadaver dissections [54]. Anatomical dissections by Lykoudis [68], Whetzel [72] and Juricic [53] did not exhibit any perforators over the muscle in several of their dissections.

Although supplying a small portion of the gracilis muscle, reports have showed dominance of the minor pedicles regarding the blood supply of the fasciocutaneous flap below the proximal one third of the muscle [54], [60], [69], [72]–[74]. These minor pedicle vessels have communications between each other and with the musculocutaneous branches of the main pedicle in the proximal to middle area of the skin flap. It has been indicated that harvesting the ‘peri-gracilis’ fat and fascia would therefore preserve the fasciocutaneous blood supply to the overlying skin and prevent flap necrosis [54], [72]. There is a significant septocutaneous perforator, which is a direct branch of the superficial femoral artery travelling to the skin between the sartorius and gracilis muscles [72], [73]. Core realized the significance of this distal perforator and used it as a second microvascular arterial conduit to perfuse a free longitudinal gracilis musculocutaneous flap with an extended skin paddle, called “the turbo gracilis myocutaneous flap [74]”. Despite this knowledge, the rich subfascial vascular connections and cutaneous perforators of the minor distal pedicles have not been elucidated to the same extent as those of the main pedicle.

**Figure 2:**
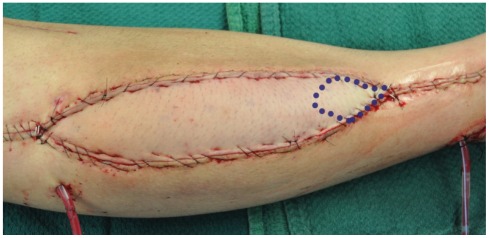
Intra-operative relative distal cutaneous ischaemia in a longitudinally designed skin flap.

Relative distal cutaneous ischaemia in longitudinally designed flaps is something many surgeons will have encountered peri-operatively **(** **figure 2** **),** which may persist and result in partial flap necrosis. The more recent transverse design of the skin paddle has improved flap viability, however exact horizontal vascular dimensions are yet to be defined. There is still information to be determined about the minor distal pedicles and their contributions. Using cadaveric dissections, angiographic and radiographic studies and advanced computer programs modified to characterize vessel sizes and densities, this current study aims to investigate the contributions of both the major and minor pedicles to the gracilis muscle and overlying fasciocutaneous component. We also investigated the route taken from the source vessel to the skin (musculocutaneous or septocutaneous) and the location and characteristics of the choke zones (joining of vascular territories) in order to optimise flap volume and blood supply.

## Methods

An anatomical study was undertaken on 27 cadaveric thigh specimens (12 fresh and 15 embalmed - standard preserving solution 10% ethanol, 30% glycerol, 60% water). The cadavers spanned a wide range of body habitus types and the cadaver ages ranged from 45 to 96 years (mean age 72 years). Institutional Ethical Approval was obtained prospectively, and conforms to the provisions of the Declaration of Helsinki in 1995. (University of Melbourne Human Ethics Committee, Ethics number HEAG 1136174). The subjects gave informed consent and patient anonymity has been preserved. All consent was written in nature, and where deceased, written consent was obtained from the next of kin of the deceased.

The neurovascular anatomy of the gracilis muscle and its associated skin paddle were investigated through a combination of dissection, microdissection and injection techniques, and radiographic studies. Digital photography was performed using a Sony Cyber-Shot 10 Megapixel Digital Camera.

The gracilis musculocutaneous flaps were raised with incisions inferiorly at the level of the tibial condyle, a longitudinal incision in line with the ASIS, continued along the groin crease towards the inferior border of the pubis, raising a flap larger than traditional maximal dimensions to study the extent of the gracilis vascular territories. The flap was raised from medially to laterally to reach the medial border of adductor longus, exposing the main gracilis vascular pedicle and nerve entering gracilis on its deep surface. The distal minor gracilis pedicles were identified by reflecting Sartorius laterally. All musculo- and fasciocutaneous branches were noted and traced back to their origin. The anatomical and morphological parameters of the gracilis muscle and neurovascular structures (origin, diameter, magnitude and location) were recorded before the pedicles were cut at their origin from the source vessel.

Each dissection was conducted by the same author (MK),with dissection times improving over the course of the dissections from mean 45 minutes for the first three dissections, and decreasing to a mean of 20 minutes over the course of the study (range 15–45 minutes). This time comprised the total time from initial incision to identification of all vascular pedicles and cutaneous perforators.

Only 25 of the 27 flaps were prospectively considered large enough for radiographic analysis. In these 25 larger gracilis musculocutaneous flaps, the major and minor gracilis pedicles were cannulated using an appropriately sized cannula and flushed with 6 % hydrogen peroxide (Orion Laboratories Pty. Ltd., Western Australia, Australia) before being selectively injected using a lead oxide/ milk powder mixture (P_3_O_4_ Red Lead; Ajax Chemicals, Australia / milk powder: Nurture, Toddlers; Heinz Ltd., Victoria, Australia) in a water suspension until resistance was experience. Specimens were left in a cool room at 4°C overnight to set. One specimen was harvested after a total lower limb injection via the common iliac artery using a Foley catheter.

Following imaging using plain radiography (Fuji Computed Radiography Processor, Model CR-IR 357, Fuji Film Corp., Tokyo, Japan), the layers of the compound flap were sequentially separated to evaluate the vasculature in each layer: 1) deep component of gracilis fascia, 2) gracilis muscle, 3) superficial layer of gracilis fascia and adductor longus fascia, 4) skin and superficial fascia and fat. Radiographic studies were performed between each removal stage and images analysed using Adobe Photoshop CS5 to colour match the corresponding pedicles and vascular territories.

The radiographic injection studies of both the gracilis muscle and overlying fasciocutaneous component were opened in ‘Image J**®**’, and the image ‘initialized’ in the previously published ‘Lymphatic Vessel Analysis Protocol’ (LVAP) plug-in [75]. Images were overlaid with two grids using the insert grid plug-in. The grids used were 200-pixel square grid with 4 pixel line width; and a 20-pixel square grid with 1 pixel line width. The muscles and fasciocutaneous tissues were divided into upper, middle and lower thirds. To commence quantification the blinded operators visualized each one third of the gracilis muscle in a viewing window at 200% zoom. A parameter was then selected using the LVAP settings panel. Aided by the grid, quantifiers proceeded systematically through each one third of the image. The gracilis muscle and the fasciocutaneous tissues overlying the muscle were quantified separately. Parameters quantified were: 1) vessel density in each one-third zone; 2) vessel caliber in each one-third zone; 3) area of tissue supplied by each perforator (perforator angiosome / perforasome area). Data were integrated into an Excel spreadsheet for statistical analysis. Averaged field counts for each parameter were collated for all images and used to obtain averages for each zone assessed, before data for all zones of all images were de-identified and grouped to generate averages for each area of each tissue. Statistical significance was determined via the Student’s T test (Microsoft Excel).

## Results

### Vascular Pedicles

The origins of the major and minor pedicles and their morphological characteristics are presented in **Table 2** **.** A double main pedicle was seen in 3 of the 27 cases, where both pedicles had separate origins from the profunda femoris vessels and were of equal size. Distal to the main pedicle, there were either one (7/27 cases), two (14/27 cases) or three (6/27 cases) minor pedicles (mean, 1.7 minor pedicles per muscle). A proximal accessory pedicle was observed in 7 cases.

**Table 2:** A summary of the anatomical characteristics of the gracilis vascular pedicles.

Pedicle	Location (muscle)	Origin	Distance from pubic attachment (cm)	Caliber (mm)	Length (cm)
Main	Proximal third	PF - 25 cases; MCFA - 2 cases	8–13	0.8–2.5	5.5–11
First minor	Middle third	SFA	15–28	0.8–1.5	3–9
Second minor	Middle/distal third	SFA - 10 cases; PA - 2 cases	25–35	0.7–1.0	2–5
Third minor	Distal third	SFA	30–36	0.5–1.0	2–5
Proximal accessory	Proximal	OA - 4 cases; AB - 2 cases; PF - 1 case		0.5–1.0	4.5–11

PF (profunda femoris), MCFA (medial circumflex femoral artery), SFA (superficial femoral artery), PA (popliteal artery), OA (obturator artery), AB (ascending branch of main pedicle).

### Perforators

A total of 112 cutaneous perforators were found, with an average of 7.5 perforators per thigh. Musculocutaneous perforators were more numerous than their septocutaneous counterpart (85 vs. 27), particularly in the proximal third of the muscle in close proximity to the area where the main pedicle entered the gracilis. The less numerous septocutaneous perforators had a larger calibre and predominated in the distal two-thirds of the muscle. The majority (20/27) septocutaneous perforators were measured at 1 mm in diameter, whereas all musculocutaneous perforators were found to be less than this diameter (**Table 3**). 17 of these vessels branched directly from the first minor pedicle.

**Table 3:** A summary of the anatomical characteristics of the gracilis perforator vessels.

Location (muscle)	Number	Musculo-cutaneous	Septo-cutaneous	Main perforator vessel	Caliber of main perforator vessel (mm)
Proximal third	57	50	7	Musculocutaneous	0.8
Distal two-thirds	55	35	20	Septocutaneous	1.0

**Figure 3:**
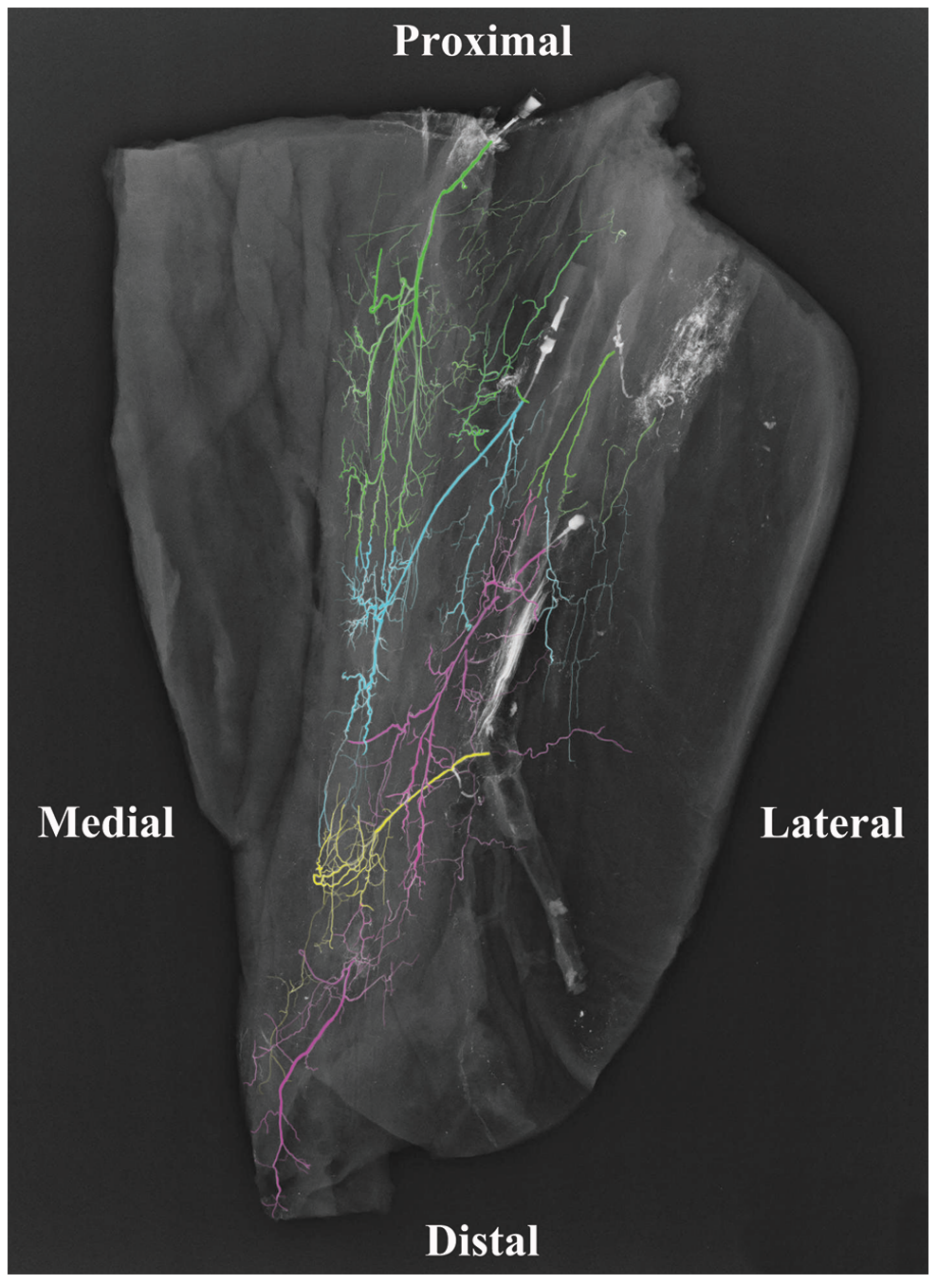
Angiogram of a cadaveric gracilis musculocutaneous flap showing the intramuscular arterial branching pattern of each pedicle. (Green: Main pedicle, Blue: First minor pedicle, Pink: Second Minor Pedicle, Yellow: Third minor pedicle.).

The intramuscular vascular arrangement was explored via microscopic dissection after the gracilis had been removed from the adipofasciocutaneous flap. The bright colouring of the vessels after injection simplified this dissection. The vascular network comprised vessels running longitudinally proximally and distally, parallel to the muscle fibres, with some interconnections between these branches. It was noted that the main pedicle divided into 2 to 5 branches, upon its entry into the muscle, travelling uniformly as transverse, ascending and descending branches. Radiographic evaluation was required to further investigate the intramuscular inter-pedicle anastomoses. **Figure 3** shows a three territory flap where there was a choke zone located between the descending branches of the main pedicle and ascending branches of the first minor pedicle (choke zone 1) in the middle third of gracilis and between branches of the first and second minor pedicles (choke zone 2) in the distal third of gracilis. Some cadavers were only had two territories (one choke zone). The main pedicle supplied the bulk of the muscle.

### Nerve anatomy

Motor innervation of the gracilis muscle is provided by the anterior branch of the obturator nerve. The diameter of this ‘nerve to gracilis’ was relatively consistent, with a mean length recorded at 10.2 cm (range, 7.5 to 12 cm) and mean diameter at 2.1 mm (range, 1 to 4 mm).

### Fasciocutaneous Blood Supply

Of the twenty-five radiographic studies conducted following lead oxide injection, only 12 were successful and used in the analysis. The remaining 13 studies could not be analysed due to the rigidity of the embalmed arterial wall or in some cases, the blood clot or atherosclerotic plaques occluding the lumen and preventing contrast perfusion of the skin flap.

Radiographic images from lead oxide injection of the main and minor gracilis pedicles showed cutaneous vascular territories that extended more posteriorly than anteriorly. The dimensions and relationships are summarized in **Tables 4** **and** **5** **.**

**Table 4:** Cutaneous vascular territory of the gracilis flap based on lead oxide injection into the dominant/main vascular pedicle.

Specimen	Overall dimensions (L×W)	Area of main pedicle cutaneous territory (cm^2^)	Extent of perfusion towards anterior thigh from pedicle entry (cm)	Extent of perfusion towards posterior thigh for pedicle entry (cm)	Extent of perfusion proximally from pedicle entry (cm)	Extent of perfusion distally from pedicle entry (cm)
**1**	15×13	195	4.5	9	5	11
**2**	32.5×16	520	7.5	9	6	28.5
**3**	33.5×10	335	3.5	7.5	5.5	29
**4**	33×19.5	643.5	6	13	7	26
**5**	35×19	665	6	13	6	30
**6**	18×10	180	4	7	7	12
**7**	34×11.5	391	4	8.5	6	7
**8**	18.5×13	240.5	6	8	4.5	15
**9**	33.5×16	536	6.5	10.5	6.5	28
**10**	22×14	308	5	10	7	16
**11**	30×18	540	7	11	6	24
**12**	26×9	234	4	6	7	20
**Mean**	27.5×14	399	5.3	9.4	6.1	20.5
**Range**	15×13 to 35×19	180 to 665	3.5 to 7.5	6 to 11	4.5 to 7	7 to 29

**Table 5:** Cutaneous vascular territory of the gracilis flap based on lead oxide injection into the first minor vascular pedicle.

Specimen	Overall dimensions(L×W)	Area of minorpedicle cutaneousterritory(cm^2^)	Extent of perfusion on anterior thigh from pedicle entry (cm)	Extent of perfusionon posterior thighfor pedicleentry (cm)	Extent of perfusion proximally from pedicle entry (cm)	Extent of perfusion distally from pedicle entry (cm)
**1**	15×9	135	3	6.5	12	4
**2**	32.5×10.5	341.25	4	7.5	21	12.5
**3**	33.5×9	301.5	3	6	15.5	18
**4**	33×12	396	4	8	25.5	9.5
**5**	35×9	315	3	7	26	10
**6**	27×8	216	3	6	14	14
**7**	34×10	340	3	8	12	12
**8**	26.5×8	212	2.5	6.5	12.5	15
**9**	33.5×10.5	351.75	4	7.5	20.5	14
**10**	29×8	232	3	5	14	15
**11**	30×11	330	3.5	8.5	21	10
**12**	35×5.5	192.5	3	3.5	15	21
**Mean**	30.3×9.2	280.25	3.25	6.7	16.9	12.3
**Range**	15×9 to 34×10	135 to 396	2.5 to 4	3.5 to 8.5	5.5 to 26	4 to 21

The ‘Lymphatic Vessel Analysis Protocol’ (LVAP) plug in for Image J®, previously described by Shayan et al.^75^ used to quantitatively analyze the distribution of the blood supply (both density and vessel caliber) showed interesting trends in both the muscle and the fasciocutaneous components.

**Figure 4:**
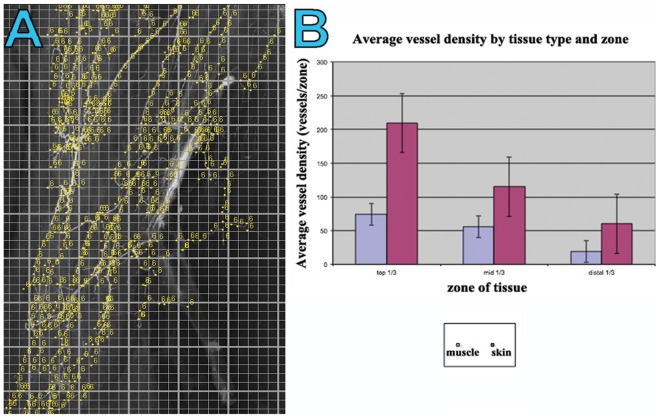
(A) A ‘screen grab’ image demonstrating the process of quantifying a radiographic injection study of the gracilis muscle and overlying fasciocutaneous tissues. Image was opened in Image J**®** and ‘initialized’ in the LVAP ‘Lymphatic Vessel Analysis Protocol’ (LVAP) plug-in. The image was then overlayed with two grids (see methods) to allow systematic quantification of vessel density. Each yellow dot represents a counting point. (B) Graphical representation of the quantified average vessel density in both the muscle and skin specimens, in each one third of the flap.

**Figure 5:**
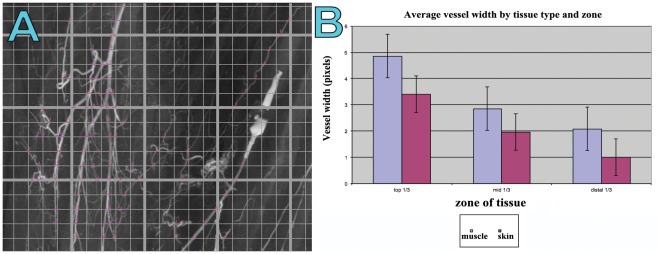
(A) A ‘screen grab’ image demonstrating the process of quantifying a radiographic injection study of the gracilis muscle and overlying fasciocutaneous tissues. Image was opened in Image J**®** and ‘initialized’ in the LVAP ‘Lymphatic Vessel Analysis Protocol’ (LVAP) plug-in. The image was then overlayed with two grids (see methods) to allow systematic quantification of vessel density. A mouse click is made at the commencement of a vessel as it crosses a grid line and a second click on opposite side of the same vessel. The LVAP program then measures the intervening distance as vessel width for the given vessel, and all widths for the viewing screen (representing one third of the specimen) are averaged and collated. (B) Graphical representation of the quantified average vessel width in both the muscle and skin specimens, in each one third of the flap.

**Figure 6:**
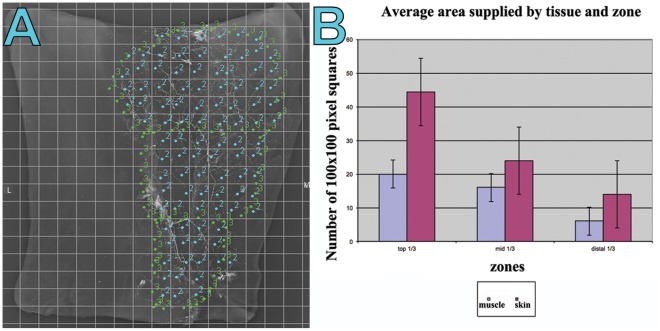
(A) A ‘screen grab’ image of a radiographic injection study of the gracilis muscle and overlying fasciocutaneous tissue demonstrating the relative perfusion for each one third of the tissues. Image opened in Image J**®** and ‘initialized’ in the LVAP ‘Lymphatic Vessel Analysis Protocol’ (LVAP) plug-in. (B) Graphical representation of the relative perfusion for each one third of the tissues of the gracilis muscle and overlying fasciocutaneous tissues.

Graphical comparison demonstrated a step-wise decrease in the average vessel density from the upper to middle and lower thirds of both the gracilis muscle and the overlying skin paddle (**Figures 4a & 4b**). Overall, there was a significantly higher average vessel density in the skin compared to the muscle. In contrast, the average vessel width was greater in the muscle than in the overlying skin **(** **Figures 5a & 5b** **)**. Comparison of vessel width between the respective zones of both the muscle and skin paddle demonstrated a progressively decreasing average vessel width from the upper to middle and lower third zones. The relative areas of blood supplied by each pedicle are shown in **Figures 6a & 6b**, clearly demonstrating the dominance of the proximal pedicle over the distal pedicle(s).

**Figure 7:**
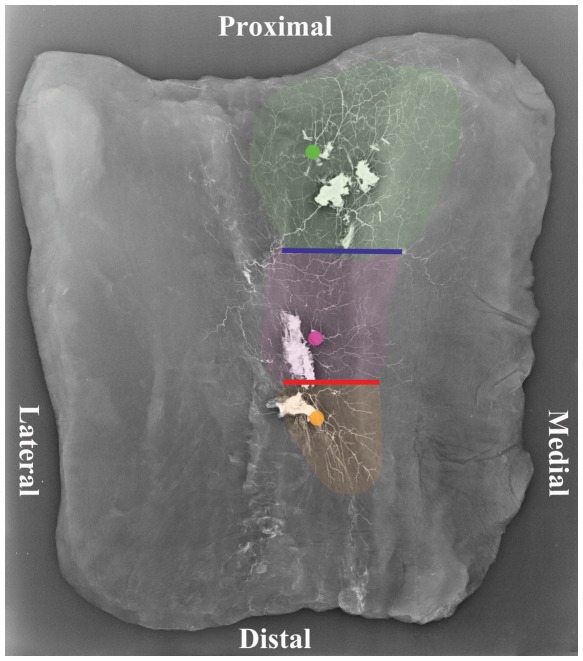
An angiogram of a cadaveric gracilis musculocutaneous flap showing a ‘three territory’ flap. (Green dot: Main pedicle, Purple dot: First minor pedicle, Orange dot: Second Minor Pedicle, Yellow: Third minor pedicle.) The blue line signifies ‘choke zone 1’ and the red line ‘choke zone 2.’

**Figure 8:**
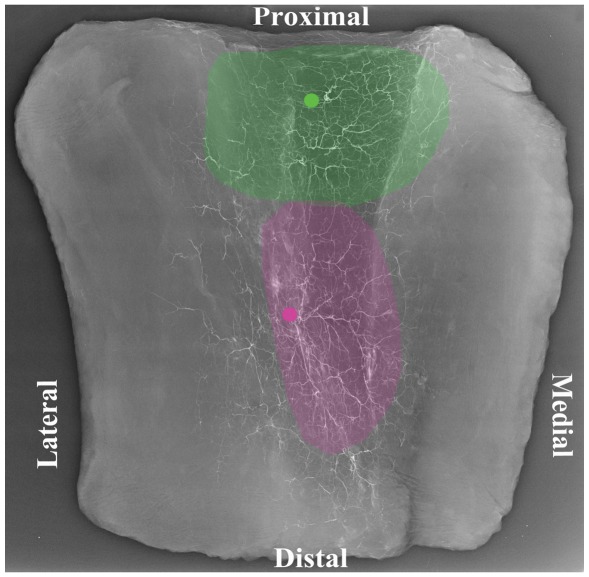
A cadaveric angiogram showing the gracilis cutaneous ‘two territory’ flap taking the shape of a “T”, positioned posteriorly.

The main pedicle perforator branches anastomosed with ascending perforator branches of the first minor pedicle at ‘choke zone 1’. This zone lay between 8.5 and 29 cm from the groin crease (mean, 15.4 cm). If a second minor pedicle was present (representing a 3 territory flap), the descending perforator branches of the first minor pedicle anastomosed with the ascending branches of this smaller distal pedicle ‘choke zone 2’, lying between 20.5 and 22 cm from the medial groin crease (mean, 21 cm) **(** **Figure 7** **)**. The third minor pedicle, if present, did not have a cutaneous contribution because of its distal position at the musculotendinous junction. The entire gracilis cutaneous territory took the shape of a “T”, positioned posteriorly **(** **Figure 8** **)**. All proximal perforators originated within 7×6 cm^2^ area from the entrance point of the main pedicle. The distal perforators were located within a 4×3 cm^2^ area from the entrance point of the minor pedicle.

## Discussion

The confusion of the cutaneous angiosomes of the gracilis musculocutaneous flap has been reported as the major contributing factor to cutaneous necrosis, and propagates its unreliability [34], [53], [76]. In contrast to previous studies which suggest that the reliable cutaneous territories only depend on the main pedicle [55], [63], [76], [77], we present novel observational and quantitative information to show that both major and minor pedicles have a significant contribution to the fasciocutaneous arterial network. These findings have clinical relevance in relation to the safety and versatility of the gracilis flap, and when combined with modern technologies such as computed tomographic angiography and the use of surgical delay in selected cases, will significantly enhance flap planning, increase the size of reconstruction possible, and improve outcomes.

**Figure 9:**
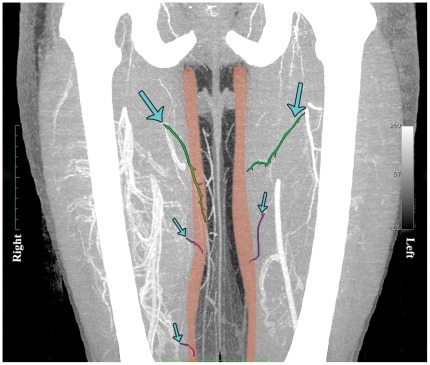
A computed tomographic angiogram (CTA) of the lower limbs, with coronal maximum intensity projection (MIP) reformat, highlighting the gracilis muscle and its major and minor arterial muscular pedicles. On the left, one major (green) and one minor (purple) pedicle can be seen and on the right, one major (green) and two minor (purple) pedicles. (Right thigh ‘3 territory’ flap and left ‘two territory’ flap).

### Dissection Studies

Broadly speaking our study showed morphological characteristics of the vascular pedicles to the gracilis to be comparable to the previous literature [55], [60], [61], [64], [67], [71], [78]. The main pedicle in our study originated from the profunda femoris in almost all our dissections (25/27), similar to the findings of the key papers by Morris [60] and Yousif [54]. Other authors have found that the origin was from the medial circumflex femoral artery [29], [61], [67]. The dimensions and entry point of the main pedicle into the muscle also correlated with previous work [54], [60], [79], [80]. The number and origin of the minor pedicles in our study was comparable to the work of Taylor [65] and Morris [60], with the exception of two cases where the second minor pedicle arose from the popliteal vessels. The first minor pedicle was consistent. The ‘accessory pedicles’ we found (7/27), proximal to the main pedicle, previously described by Taylor [65], in practice are not clinically relevant as they had no cutaneous contributions.

**Figure 10:**
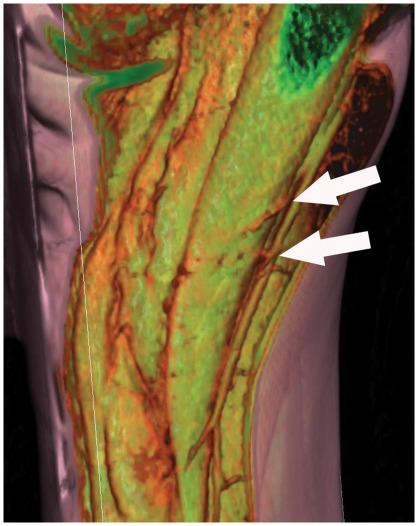
A volume rendered (VR) reformat of a computed tomographic angiogram showing musculo-cutaneous perforators traversing the gracilis muscle to supply the overlying skin. (Reproduced with permission from: Rozen WM, Chubb D, Grinsell D, Ashton MW. *Computed tomographic angiography: clinical applications*. Clin Plast Surg. 2011 Apr; 38(2): 229–39.).

Musculocutaneous perforators were dominant in number throughout the muscle, particularly in the proximal third. They always exited the muscle close to the main pedicle entry point as confirmed by prior studies [54], [55], [63], [76]. It is difficult to understand how Lykoudis [68] and Juricic did not find similar perforators [53]. The septocutaneous perforators were larger in diameter (average 1 mm), with the majority originating from the first minor pedicle. Our findings infer that only a small cuff of muscle may be required to preserve adequate vasculature when taken with a proximally based gracilis fasciocutaneous flap. Conversely, with septocutaneous perforators residing along the middle and distal thirds of the muscle and due to their large calibre, it may be sufficient to base a longitudinal middle based fasciocutaneous flap without sacrificing the muscle. If a longer flap is required, the first minor pedicle may be taken with a muscle cuff (at least 4×3 cm) to preserve the musculocutaneous perforators. In spite of the important contribution from the minor pedicle(s) found, there was a larger average area of cutaneous perfusion originating from the main pedicle, with mean dimensions of 27.5×14 cm (399×cm^2^), as opposed to the minor pedicle, with mean dimensions 30.3×9.2 cm (280×cm^2^).

### Radiographic Studies and Quantitative Evaluation

Our radiographic injection studies demonstrate either two (**Figure 8**) or three (**Figure 7**) vascular territories in the fasciocutaneous component of the gracilis flap, supplied by the main and minor gracilis pedicles. The intramuscular branches of these pedicles ran longitudinally, anastomosing with each other at choke zones 1 and 2. The computerised quantification of both the gracilis muscle and overlying fasciocutaneous tissue gives an interesting insight into the relative reliabilities of each component. The muscle had a higher average vessel width in each of the thirds studied. The bar charts show clearly the vessel width decreasing from the proximal to middle and distal thirds (**Figure 5b**). The vessel density however was much higher in the fasciocutaneous component, particularly in the proximal third (**Figure 4b**). The zones (area) of blood supply showed a similar relationship (**Figure 6b**).

These injection studies support the notion that the presence of choke zones are important when considering the potential length of the skin paddle. Adhering to the angiosome concept [81], maintaining the choke vessel interface between the main and minor pedicles (choke zone 1) would allow a flap based on just the main pedicle. In some instances this could supply a skin flap of up to 35×19 cm. This could be an under estimation as Saint Cyr’s work suggests cadaveric injection studies underestimate the perfusion of tissue in vivo82. It makes sense, considering our quantitative assessment and characteristics of the blood supply to each third, that in the presence of a second choke zone (choke zone 2), ie: a three territory flap, that necrosis of the distal component is likely.

### Implication of Findings for Breast Reconstruction

Our anatomical studies show that, for large reconstructions, if a ‘T’ or tri-lobed flap with an extended vertical component is needed, it is important to establish if three territories (that of the main, first minor and second minor pedicles) including both choke zone 1 and 2 are present. To safely plan a gracilis myocutaneous free flap, especially for a large breast reconstruction, incorporating both a transverse and an extended vertical component, it would be important to know how many choke zones were present. Modern technologies such as computed tomographic angiography offers this exciting possibility. The surgeon may pre-operatively visualise the number and position of major and minor pedicles which can vary from left to right in the same individual. **Figure 9** shows a computed tomographic angiogram (CTA) of the lower limbs, with coronal maximum intensity projection (MIP) reformat, highlighting the gracilis muscle and its major and minor arterial muscular pedicles. On the left one major (green) and one minor (purple) pedicle can be seen and on the right, one major (green) and two minor (purple) pedicles. Therefore the right thigh would be a 3 territory flap and the left a two territory flap. It is possible to raise the right flap without necrosis by using surgical delay [83].This would involve surgical division of the first minor pedicle. The work of Dhar and Taylor [83] suggests this should be done a minimum of 72 hours prior to definitive flap transfer. If delay is not possible, and the entire gracilis flap is required, then pre-operative imaging would allow the choice of the left thigh (2 territory flap) instead of the right as donor site.

Gracilis flaps based solely on the minor pedicles are rare [66], and to our knowledge have included muscle and only been used for upper and lower limb reconstruction thus far. Our findings support the concept that small volume breast reconstructions can be performed on suitable patients, based on septocutaneous perforators from the minor pedicle without the need to harvest any muscle, further reducing donor site morbidity. The reliability of this procedure may also be improved with the use of computed tomographic angiography. **Figure 10**, a volume rendered (VR) reformat of a computed tomographic angiogram, highlights musculo-cutaneous perforators traversing the gracilis muscle to supply the overlying skin, to show that this patient would not be suitable for such a procedure.

As such, for the reconstruction of small to medium sized breasts, the traditional transverse musculocutaneous gracilis flap (TMG) [30], [37], [54], which has minimal functional donor-site morbidity [84] is a safe and reliable option. Extended TMG variants as described by several authors [57], [82] are capable of yielding flaps up to 576 g and skin paddles of 30 cm×10 cm [28]. The tri-lobed flap, recently suggested following an elegant study by Saint-Cyr [26], [82] building on the previous work of Peek [55] and Vega are exciting alternatives. Extended longitudinal components, either in isolation or as part of a ‘tri-lobed or T flap’, however, risk potentially including more than two territories (perforasomes [85] or perforator angiosomes [86]) and may result in an unreliable distal component. We believe the results of this study and other recent work [82], combined with non invasive pre operative imaging such as computed tomographic angiography (CTA) [87] and potentially magnetic resonance angiography (MRA) [88] will help predict the number of vascular territories due to pedicle arrangements, and aid in safe and reliable flap design and optimal patient satisfaction. Precise pre-operative planning may also therefore allow small breast reconstructions based on a minor gracilis pedicle, with no functional donor site morbidity.
